# Antibiotic Resistance and Antibiotic Resistance Genes in *Escherichia coli* Isolates from Hospital Wastewater in Vietnam

**DOI:** 10.3390/ijerph14070699

**Published:** 2017-06-29

**Authors:** La Thi Quynh Lien, Pham Thi Lan, Nguyen Thi Kim Chuc, Nguyen Quynh Hoa, Pham Hong Nhung, Nguyen Thi Minh Thoa, Vishal Diwan, Ashok J. Tamhankar, Cecilia Stålsby Lundborg

**Affiliations:** 1 Health Systems and Policy (HSP): Improving the Use of Medicines, Department of Public Health Sciences, Karolinska Institutet, Tomtebodavägen 18A, 17177 Stockholm, Sweden; vishaldiwan@hotmail.com (V.D.); ejetee@gmail.com (A.J.T.); Cecilia.Stalsby.Lundborg@ki.se (C.S.L.); 2 Department of Pharmaceutical Management and Pharmaco-Economics, Hanoi University of Pharmacy, 13-15 Le Thanh Tong, Hoan Kiem District, Hanoi 110403, Vietnam; 3 Department of Dermatology and Venereology, Department of Family Medicine, Department of Microbiology, Hanoi Medical University, 01 Ton That Tung, Dong Da District, Hanoi 116516, Vietnam; landhy2003@yahoo.com (P.T.L.); ntkchuc@yahoo.com (N.T.K.C.); hongnhung@hmu.edu.vn (P.H.N.); ntmthoa2017@gmail.com (N.T.M.T.); 4 National Centralized Drug Procurement Centre, Vietnam Ministry of Health, 138A Giang Vo Street, Ba Dinh district, Hanoi 118401, Vietnam; quynhhoa29@gmail.com; 5 Department of Microbiology, Bach Mai Hospital, 78 Giai Phong, Dong Da District, Hanoi 116365, Vietnam; 6 Department of Public Health & Environment, R.D. Gardi Medical College, Agar Road, Ujjain 456006, India; 7 Indian Initiative for Management of Antibiotic Resistance, Department of Environmental Medicine, R.D. Gardi Medical College, Agar Road, Ujjain 456006, India

**Keywords:** antibiotic resistance, antibiotic resistance genes, *bla_CTX-M_*, *bla_TEM_*, *qepA*, hospital wastewater

## Abstract

The environmental spread of antibiotic-resistant bacteria has been recognised as a growing public health threat for which hospitals play a significant role. The aims of this study were to investigate the prevalence of antibiotic resistance and antibiotic resistance genes (ARGs) in *Escherichia coli* isolates from hospital wastewater in Vietnam. Wastewater samples before and after treatment were collected using continuous sampling every month over a year. Standard disk diffusion and E-test were used for antibiotic susceptibility testing. Extended-spectrum beta-lactamase (ESBL) production was tested using combined disk diffusion. ARGs were detected by polymerase chain reactions. Resistance to at least one antibiotic was detected in 83% of isolates; multidrug resistance was found in 32%. The highest resistance prevalence was found for co-trimoxazole (70%) and the lowest for imipenem (1%). Forty-three percent of isolates were ESBL-producing, with the *bla_TEM_* gene being more common than *bla_CTX-M_.* Co-harbouring of the *bla_CTX-M_*, *bla_TEM_* and *qepA* genes was found in 46% of isolates resistant to ciprofloxacin. The large presence of antibiotic-resistant *E. coli* isolates combined with ARGs in hospital wastewater, even post-treatment, poses a threat to public health. It highlights the need to develop effective processes for hospital wastewater treatment plants to eliminate antibiotic resistant bacteria and ARGs.

## 1. Introduction

The environmental spread of antibiotic resistant bacteria has been recognized as a growing public health threat [1,2]. Hospitals are “hotspots” for antibiotic use and not only play an important role in antibiotic dissemination but also in the release of antibiotic resistant bacteria into the environment. Hospital wastewater treatment plants containing antibiotic residues can favour the development of antibiotic resistance due to the selective pressure placed on bacteria [3,4]. Moreover, antibiotic resistance genes (ARGs) carried by bacterial contaminants can be transferred to other bacterial populations including pathogenic bacteria found in hospital wastewater [1]. Hospital effluents can reach water bodies used in agriculture or for domestic purposes. From there, antibiotic resistant bacteria and/or ARGs can be transferred to humans.

In recent years, the presence of antibiotic resistant *Escherichia coli* (*E. coli*), particularly extended-spectrum beta-lactamase (ESBL)-producing isolates, in surface water has attracted attention [5]. A direct relationship between clinical *E. coli* isolates and the quantity of ESBL-producing *E. coli* strains found in hospital wastewater has been demonstrated [6]. Consequently, the existence of ESBL-producing *E. coli* carriers in hospitals may lead to their environmental spread [6].

Antibiotic resistance causes prolonged illness, excess mortality, and higher costs for patients and health systems [7,8,9]. Despite increased warnings and numerous efforts to contain it, antibiotic resistance has been increasing [10,11,12,13]. At the recent United Nations general assembly, it was highlighted that antibiotic resistance is among the greatest global health risks, requiring urgent attention [14].

The risks are potentially more serious in low- and middle-income countries where many hospitals either do not have wastewater treatment plants or they are ineffective. To make matters worse, in many places, but particularly rural areas, surface water is used for agriculture and domestic purposes or even consumed untreated. Most research on antibiotic resistant bacteria in hospital wastewater originates from high-income countries [15].

Therefore, this study sought to investigate the prevalence of resistant *E. coli* isolates to commonly used antibiotics, ESBL-producing isolates along with genes coding for cephalosporin resistance *bla_CTX-M_* and *bla_TEM_*, and a gene coding for ciprofloxacin resistance *qepA*, in hospital wastewater in a rural and an urban hospital in Vietnam.

## 2. Materials and Methods

This is a repeated cross-sectional study with monthly data collection in one rural and one urban hospital in Vietnam, a lower middle-income country. The rural hospital has 220 beds and is situated 60 km northwest of central Hanoi. The 520-bedded urban hospital is located in central Hanoi. Both hospitals’ wastewater is routed to wastewater treatment plants (WWTPs) where it is treated using filtering, microbiological, and biochemical mechanisms. After treatment, hospital effluents are discharged into sewer systems, which lead to nearby rivers.

### 2.1. Collection of Water Samples

The collection of water samples is described in detail elsewhere [4]. Briefly, samples of wastewater before treatment (WBT) as well as wastewater after treatment (WAT) were collected using 24 h continuous sampling on a weekday during the last week of every month in 2013. The water samples were stored in closed containers surrounded by ice and transferred to the microbiological laboratory in Bach Mai Hospital in central Hanoi within 6 h of testing. The urban hospital and its wastewater treatment plant was under reconstruction from June to August 2013, therefore sampling was ceased during this period.

### 2.2. Antibacterial Susceptibility Testing and Detection of ARGs

Coliforms were detected with the most probable number procedure [16]. A presumptive test involved three subsets of tubes containing different amounts of lactose or lauryl tryptose broth. Each subset contained five tubes with inverted Durham tubes to collect gas produced by fermentation. The three subsets were inoculated with water samples of 10, 1.0, and 0.1 mL, respectively. The tubes were then incubated for 24 h at 35–37 °C. A positive test for gas formation was presumptive evidence of coliforms. A confirmatory test for coliforms was made by inoculating another broth from one of the positive tubes. The test was completed by final isolation of the coliforms on selective and differential media, Gram staining the isolates, and reconfirming gas production. Coliform isolates were then sub-cultured on *Brilliance*^TM^ UTI agar to collect presumptive *E. coli* isolates. Following biochemical confirmation using standard tests, identified *E. coli* isolates were tested for antibiotic susceptibility using the standard Kirby Bauer disc diffusion method for: (i) amoxicillin/clavulanic acid; (ii) ceftazidime; (iii) ceftriaxone; (iv) ciprofloxacin; (v) co-trimoxazole (trimethoprim/sulfamethoxazole); (vi) fosfomycin; (vii) gentamicin; and (viii) imipenem. The selected antibiotics were commonly used in the hospitals and routinely tested in clinical laboratories. Antibiotic susceptibility test results were interpreted as resistant, intermediate, and susceptible using Clinical and Laboratory Standard Institute guidelines (CLSI M100-2013) [17]. Minimum inhibitory concentrations (MICs) were determined for ciprofloxacin and ceftazidime using E-test. Disc diffusion zone diameters were also compared with epidemiological cut-off values (ECOFF) [18].

For *E. coli* isolates resistant to third generation cephalosporins, extended-spectrum beta-lactamase (ESBL) production was tested using combined disc diffusion. Genotypic confirmation was done through polymerase chain reactions. Genes coding for beta-lactam resistance, *bla_CTX-M_* and *bla_TEM_*, were tested in ESBL-producing isolates and *qepA* gene was tested for in ciprofloxacin-resistant isolates [19,20].

### 2.3. Data Analysis

Prevalence of resistance to at least one of the studied antibiotics, to each studied antibiotic, and multidrug resistance (MDR) in *E. coli* isolates were analysed. The definition of MDR reported by Magiorakos et al. (2012) from ECDC Joint Expert Meetings was applied; according to that, bacterial isolates were considered multidrug-resistant if they were non-susceptible to at least one agent in three or more antibiotic categories [21]. Fisher’s exact test was applied to test the difference between the prevalence of antibiotic resistant *E. coli* isolates before and after wastewater treatment in each hospital using Stata 12 (StataCorp LP, College Station, TX, USA).

## 3. Results

In total, 265 *E. coli* isolates were collected from both the hospitals during the study period; 158 from the rural hospital (WBT = 84; WAT = 74) and 107 isolates from the urban hospital (WBT = 60; WAT = 47).

### 3.1. Resistance to Studied Antibiotics

In the rural hospital, 85% of *E. coli* isolates were resistant to at least one of the tested antibiotics (WBT = 94%; WAT = 74%). Resistance was most common towards co-trimoxazole, with 70% of isolates being resistant to it (WBT = 86%; WAT = 53%). Resistance to ceftriaxone was found in 49% of isolates (WBT = 55%; WAT = 42%), and resistance to ceftazidime, gentamicin, and amoxicillin/clavulanic acid was around 40%, respectively. Thirty percent of the isolates were resistant to ciprofloxacin (WBT = 25%; WAT = 35%), and 2% were resistant to fosfomycin (WBT = 1%; WAT = 3%). Resistance to imipenem was only detected in one isolate (1%). MDR was found in 35% of isolates (WBT = 44%; WAT = 26%). Prevalence of resistance to the studied antibiotics in *E. coli* isolates from WAT was less common than WBT, with the exception of ciprofloxacin and fosfomycin, for which enrichment of resistant *E. coli* isolates after wastewater treatment was found. The differences are statistically significant for amoxicillin/clavulanic acid, co-trimoxazole, gentamicin, resistance to at least one studied antibiotic, and MDR (Table 1).

In the urban hospital, 79% of *E. coli* isolates were resistant to at least one of the studied antibiotics (WBT = 88%; WAT = 68%). Co-trimoxazole resistance was again most common, with resistance found in 71% of isolates (WAT = 80%; WAT = 60%), followed by ceftriaxone resistance (39%) (WBT = 45%; WAT = 32%). Resistance to gentamicin and ceftazidime was found in 29% and 28% of isolates, respectively, followed by amoxicillin/clavulanic acid (24%) and ciprofloxacin (21%). Fosfomycin resistance was least common, as it was detected in only 8% of isolates. MDR was found in 27% of isolates (WBT = 32%; WAT = 21%). Prevalence of resistance to the studied antibiotics in *E. coli* isolates from WAT was lower than WBT. The differences were statistically significant for co-trimoxazole, fosfomycin, and resistance to at least one studied antibiotic (Table 1).

**Figure 1:**
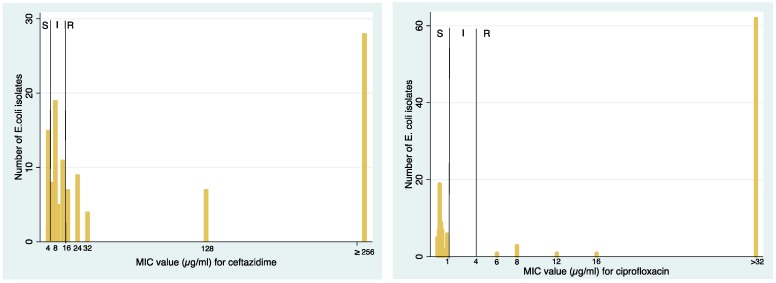
Distribution of minimum inhibitory concentration (MIC) values for ceftazidime and ciprofloxacin susceptibility testing.

The distribution of MIC values for ceftazidime and ciprofloxacin susceptibility testing is presented in Figure 1. The number of *E. coli* isolates with high MIC values is large compared to the number of isolates with lower MIC values, indicating high levels as well as high proportions of resistance.

When applying ECOFF values, we found decreased susceptibility to amoxicillin/clavulanic acid (45% of isolates), ceftazidime (39% of isolates), ceftriaxone (48% of isolates), ciprofloxacin (29% of isolates), and imipenem (2% of isolates).

MDR patterns are presented in Table 2 with identified antibiotic combinations. MDR to six out of eight studied antibiotics was found in 25 isolates (10%).

### 3.2. ESBL-Producing E. coli, ESBL, and Quinolone Resistance Genes

In the rural hospital, 76 *E. coli* isolates were ESBL-producing (48%). Among them, *bla_TEM_* was detected in 97% of isolates and *bla_CTX-M_* in 76%. Both *bla_CTX-M_* and *bla_TEM_* were detected in 75% of isolates. Quinolone-resistance gene (*qepA*) was detected in 72% of ciprofloxacin-resistant isolates. All three genes were detected in 51% of ciprofloxacin-resistant isolates (Table 3).

In the urban hospital, 39 *E. coli* isolates were ESBL-producing strains (36%). Among them, *bla_TEM_* was detected in 95% of isolates and *bla_CTX-M_* in 41%. Both *bla_CTX-M_* and *bla_TEM_* were detected in 41% of isolates. Quinolone-resistance gene (*qepA*) was detected in 86% of ciprofloxacin-resistant isolates. All three genes, *bla_TEM_*, *bla_CTX-M_*, and *qepA,* were detected in 36% of ciprofloxacin-resistant isolates (Table 3).

## 4. Discussion

Our novel findings show that, in Vietnam, bacteria resistant to commonly used antibiotics along with genes coding for resistance are present in hospital wastewater, even after treatment. Prior to our study, Duong et al. examined *E. coli* resistance to ciprofloxacin and norfloxacin in hospital wastewater of another Hanoian hospital and reported that *E. coli* strains isolated from WAT samples were susceptible [22]. In their study, water samples were collected over two days using grab sampling and only 15 *E. coli* isolates, including three isolates from treated water samples, were tested. Conversely, in our study, water samples were collected every month over one year using continuous sampling and a total of 265 *E. coli* isolates were tested.

In both hospitals, *E. coli* isolates were most resistant to co-trimoxazole (around 70% of isolates). Previous reports show comparatively lower prevalence rates. In 2004–2005, an Indian study indicated that 55% of enteric bacteria found in hospital wastewater were resistant to co-trimoxazole [23]. A study from Poland showed that 20% of *E. coli* isolates from hospital wastewater collected before 2013 were resistant to co-trimoxazole [24]. Co-trimoxazole is a combination of sulfamethoxazole and trimethoprim. Sulfamethoxazole, one of the first antibiotics to be developed, was put into clinical use in 1935 and trimethoprim was first used in 1962. The two antibiotics first started to be used in combination in 1968. Over the past decades, its extensive use in clinical settings to treat a variety of bacterial infections, such as urinary tract infections and respiratory tract infections, which was also the case in the studied hospitals, might explain the high occurrence of co-trimoxazole resistance [25]. Moreover, both sulfamethoxazole and trimethoprim are not readily degradable and their residues found every month over the studied period in the same hospital wastewater could favour the development of co-trimoxazole resistance in the bacteria [4].

Cephalosporin resistance was also found in higher proportions than other antibiotics investigated. Similar prevalence rates of cephalosporin resistant bacteria in hospital effluent were shown by Chagas et al. [26]. Resistance mechanisms to second and third generation cephalosporins differ, with ESBLs being the most important [27]. ESBL enzymes are capable of hydrolysing and inactivating beta-lactam antibiotics and are often plasmid-mediated [28]. The plasmid genes encoding ESBLs can be transferred between different bacterial strains (horizontal gene transfer), facilitating easy spread of antibiotic resistance within as well as between species. Moreover, plasmid-encoded ESBL-producing bacteria can show co-resistance to quinolones, aminoglycosides, and sulfonamides [29]. Consequently, infections caused by ESBL-producing bacterial strains can be difficult to treat due to the restricted amount of antibiotics left for successful treatment.

ESBLs were first isolated in the 1980s [30]. In a study conducted in an urban and rural hospital in central India, Chandran et al. reported a very high prevalence of ESBL-producing *E. coli* in the hospital wastewater (96%) [31]. In our study, the prevalence was around 40%, lower than the aforementioned study but relatively higher than the figures reported by Diwan et al. (25%), Abdulhaq et al. (25%), and Korzeniewska et al. (37%) [22,32,33]. Among genes coding for ESBL production, TEM and CTX-M are the most common [34]. Our findings indicate the presence of *bla_CTX-M_* and *bla_TEM_* in ESBL-producing strains, with the *bla_TEM_* gene being predominant. This is in accordance with the findings of Varela et al., where *bla_TEM_* was found to be the most prevalent ESBL-encoding gene, followed by *bla_CTX-M_* [35]. In contrast, Chandran et al. reported higher prevalence of *bla_CTX-M_* than *bla_TEM_* in hospital wastewater in India [31]. However, not all the *bla_TEM_* genes are responsible for ESBL, and in our study, we were not able to do further sequencing to show the frequency of the ESBL *bla_TEM_*. In addition, according to the PCR protocol used, the detected *bla_CTX-M_* were restricted to CTX-M group 1 including CTX-M-1, CTX-M-3, and CTX-M-15. Ciprofloxacin resistance was detected in our samples, with gene *qepA* coding for high proportions of the resistant strains. Similar to ESBL-coding genes *bla_CTX-M_* and *bla_TEM_*, quinolone resistance gene *qepA* is plasmid-mediated and capable of horizontal gene transfer [36]. Of note is that co-existence of *bla_CTX-M_*, *bla_TEM_*, and *qepA* was detected, genetically proving co-resistance in the bacterial strains. In our study, the prevalence of MDR found phenotypically was around 35% with the detection of co-resistance to six out of eight of the studied antibiotics.

Fosfomycin-resistance and imipenem-resistance were also detected among the *E. coli* isolates in our study. Fosfomycin has broad activity against Gram-negative and some Gram-positive bacteria. In some countries, it is recommended as one of the first-line drugs to treat uncomplicated urinary tract infections because of increasing *E. coli* resistance towards other commonly used antibiotics, such as ciprofloxacin and co-trimoxazole [37]. Imipenem, the first carbapenem developed, is used to treat infections caused by β-lactamase-producing bacteria and should be saved to treat infections not readily treated by other antibiotics [38]. High prevalence of carbapenem-resistance in clinical isolates in Vietnamese hospitals has been reported [39]. The detection of resistance to these last-line antibiotics in bacterial isolates from hospital wastewater is of concern, since this can contribute to the spread of resistance among bacterial populations in the environment.

Although there is increasing evidence of the occurrence of antibiotic resistant bacteria in the environment, there are no standardized methods for antibiotic susceptibility testing which are directly applicable to environmental samples so far [2]. Epidemiological cut-off values developed by the European Committee on Antimicrobial Susceptibility Testing (EUCAST) can be used for the interpretation of antibiotic resistance in environmental bacteria. The ECOFF values separate bacteria with acquired resistance mechanisms (non-wild type) from the wild type population (having no resistance) [15]. We found in general decreased susceptibility in the *E. coli* isolates when using ECOFF values.

The role of hospitals in the environmental release of antibiotic-resistant bacteria and ARGs has been demonstrated and has become a growing concern for public health [40,41,42,43,44,45,46,47]. Hospital WWTPs can harbour antibiotic-resistant bacteria and ARGs [48,49,50]. Antibiotic residues in WWTPs can favour the development of antibiotic resistance due to the selective pressure placed on bacteria. In our previous study, we found that antibiotic concentrations in wastewater collected from hospital WWTPs were often higher than the reported predicted no-effect concentrations for resistance selection as well as the minimum selective concentrations, meaning that the selection of antibiotic-resistant bacteria can occur [4]. Published studies have reported the enrichment of antibiotic-resistant *E. coli* in WWTPs [51,52]. Our findings show significant reductions as well as the enrichment of antibiotic-resistant *E. coli* in WAT. Resistance to at least one studied antibiotic in the *E. coli* isolates from WAT was still detected in high proportions. Consequently, certain amounts of antibiotic-resistant bacteria, along with ARGs, are released into the ambient aquatic environment. They can then enter water bodies used for agriculture, irrigation, or household purposes, which poses a threat to public health. The problem can be aggravated if hospitals do not have WWTPs and the wastewater is discharged directly into the environment, which is common practice in Vietnam as well as many other low- and middle-income countries [53]. It has been shown that the prevalence of antibiotic-resistant bacteria is significantly reduced by advanced wastewater treatment processes such as ozone, UV, and ultrafiltration [54,55]. However, even in such advanced plants, resistant bacteria are not completely removed, therefore, hospitals must invest in effective WWTPs with treatment processes that completely eliminate antibiotic-resistant bacteria.

It is plausible that the dissemination of antibiotic-resistant bacteria and ARGs in the environment can result in their transmission to humans [56], however, direct evidence for this is very scarce [57]. So far, the strongest evidence available has shown the genetic similarities between human-related bacterial strains and environmental isolates collected at exposure-relevant sites [58]. Further studies are needed to identify links between the discharge of antibiotic-resistant bacteria by hospital WWTPs, their occurrence in the ambient environment, and their acquisition by humans via environmental exposure.

Our study has some limitations. Importantly, the prevalence of antibiotic resistance in *E. coli* isolates presented here might not be representative for the whole *E. coli* population in the hospital wastewater because of the limited number of *E. coli* isolates from each water sample. Moreover, due to financial constraints, we were not able to study more antibiotics and ARGs than what we have done. Screening for *bla_SHV_* gene, which is also common in ESBL-producing *E. coli,* and genes coding for imipenem resistance would make the study more comprehensive. We were also not able to do sequencing for *bla_TEM_* genes to show the frequency of the genes encoding for ESBL. Another limitation is that data from the urban hospital were unavailable for three months, as sampling could not be carried out due to construction work at the hospital. Furthermore, a detailed description of the functioning of the WWTPs was not available for us.

## 5. Conclusions

High prevalence of antibiotic resistance and ARGs were detected in *E. coli* isolates from hospital wastewater both before and even after wastewater treatment. There is a need for inclusion and development of hospital WWTPs which are effective at eliminating antibiotic-resistant bacteria and ARGs. Further studies are needed to identify links between the discharge of antibiotic-resistant bacteria by hospital WWTPs, their occurrence in the nearby environment, and their acquisition by humans when exposed in the environment.

## Figures and Tables

**Table 1:** Prevalence of resistance to studied antibiotics in *Escherichia coli* isolates found in hospital wastewater.

Studied Antibiotics	Rural Hospital (*n* = 158)				Urban Hospital (*n* = 107)				Both Hospitals (n = 265) Overall (%)
	WBT (%) *n* = 84	WAT (%) *n* = 74	*p-*Value	Overall (%)	WBT (%) *n* = 60	WAT (%) *n* = 47	*p-*Value	Overall (%)	
**Amoxicillin/clavulanic acid**	51	24	0.001 *	39	28	19	0.36	24	33
**Ceftazidime**	42	36	0.52	39	32	23	0.40	28	35
**Ceftriaxone**	55	42	0.11	49	45	32	0.23	39	45
**Ciprofloxacin**	25	35	0.22	30	23	17	0.50	21	26
**Co-trimoxazole**	86	53	<0.001 *	70	80	60	0.03 *	71	70
**Fosfomycin**	1	3	0.60	2	15	0	0.005 *	8	4
**Gentamycin**	51	31	0.02 *	42	33	23	0.30	29	37
**Imipenem**	1	0	1.00	1	0	0	N/A	0	1
**At least one antibiotic**	94	74	0.001 *	85	88	68	0.02 *	79	83
**MDR**	44	26	0.02 *	35	32	21	0.30	27	32

MDR: multidrug resistance; N/A: not available; WBT: wastewater before treatment; WAT: wastewater after treatment. * Differences in prevalence of resistant *Escherichia coli* strains isolated from WBT and WAT are significant.

**Table 2:** Multidrug resistance patterns in *Escherichia coli* isolates found in hospital wastewater (the number of MDR isolates having the respective pattern).

MDR Pattern	Rural Hospital (*n* = 158)		Urban Hospital (*n* = 107)	
	WBT	WAT	WBT	WAT
CTX + CIP + SXT	0	0	0	2
CTX + GEN + SXT	7	0	3	0
AMC + GEN + SXT	0	0	1	0
GEN + CIP + SXT	0	4	7	1
CAZ + CTX + AMC + CIP	2	0	0	0
CAZ + CTX + AMC + SXT	2	0	0	2
CAZ + CTX + GEN + SXT	6	0	1	0
CAZ + CTX + CIP + SXT	0	0	0	1
CAZ + CTX + SXT + FOM	1	0	0	0
CTX + GEN + CIP + SXT	4	1	2	0
AMC + GEN + CIP + SXT	0	1	0	1
CAZ + CTX + GEN + CIP + SXT	2	2	3	0
CAZ + CTX + AMC + CIP + SXT	0	1	0	0
CTX + GEN + CIP + SXT + FOM	0	1	0	0
CTX + AMC + GEN + CIP + SXT	1	0	0	0
IMP + CAZ + CTX + AMC + SXT	1	0	0	0
CAZ + CTX + AMC + GEN + CIP + SXT	11	9	1	3
CAZ + CTX + AMC + GEN + CIP + FOM	0	0	1	0
Total (%)	37 (44%)	19 (26%)	19 (32%)	10 (21%)
	56 (35%)		29 (27%)	

MDR: multidrug resistance; WBT: wastewater before treatment; WAT: wastewater after treatment; AMC: amoxicillin/clavulanic acid; CAZ: ceftazidime; CIP: ciprofloxacin; CTX: ceftriaxone; FOM: fosfomycin; GEN: gentamicin; IMP: imipenem; SXT: trimethoprim/sulfamethoxazole (co-trimoxazole).

**Table 3:** Genetic analysis of extended-spectrum beta-lactamase (ESBL)-producing and ciprofloxacin-resistant *Escherichia coli* strains found in hospital wastewater.

Genetic Analysis	Rural Hospital			Urban Hospital			Both Hospitals
	WBT *n* (%)	WAT *n* (%)	Overall *n* (%)	WBT *n* (%)	WAT *n* (%)	Overall *n* (%)	Overall *n* (%)
**ESBL-producing**	45 (54)	31 (42)	76 (48)	27 (45)	12 (26)	39 (36)	115 (43)
*bla_CTX-M_*	29 (64)	29 (94)	58 (76)	14 (52)	2 (17)	16 (41)	74 (64)
*bla_TEM_*	44 (98)	30 (97)	74 (97)	27 (100)	10 (83)	37 (95)	111 (97)
*bla_CTX-M_* + *bla_TEM_*	29 (64)	28 (90)	57 (75)	14 (52)	2 (17)	16 (41)	73 (63)
**Ciprofloxacin resistance**	21 (25)	26 (35)	47 (30)	14 (23)	8 (17)	22 (21)	69 (26)
*qepA*	14 (67)	20 (77)	34 (72)	12 (86)	7 (88)	19 (86)	53 (77)
*qepA* + *bla_CTX-M_* + *bla_TEM_*	13 (62)	11 (42)	24 (51)	6 (43)	2 (25)	8 (36)	32 (46)

WAT: wastewater after treatment; WBT: wastewater before treatment.
